# A Deeper Assessment of ω3-Poly-Unsaturated Fatty Acids in Polycystic Ovary Syndrome Management. Comment on Regidor et al. Chronic Inflammation in PCOS: The Potential Benefits of Specialized Pro-Resolving Lipid Mediators (SPMs) in the Improvement of the Resolutive Response. *Int. J. Mol. Sci.* 2021, *22*, 384

**DOI:** 10.3390/ijms221810114

**Published:** 2021-09-18

**Authors:** Vittorio Unfer

**Affiliations:** System Biology Group Lab, 00161 Rome, Italy; vunfer@gmail.com

I have read with great interest the article recently published by Regidor et al. [1] about the specialized pro-resolving lipid mediators (SPMs) that derive from the metabolism of DHA and EPA, two essential ω3-poly-unsaturated fatty acids (PUFAs). The authors highlight that such SPMs help to resolve inflammatory processes by reducing proinflammatory cytokines and neutrophil recruitment, and by enhancing phagocytosis. Specifically, they suggest the use of ω3-PUFAs as a novel potential treatment in polycystic ovary syndrome (PCOS), acting on the chronic inflammatory process associated with the disease.

DHA and EPA are commonly used as dietary supplements in pathological contexts such as obesity [2], atherosclerosis [3] and diabetes mellitus [4], improving cardiovascular parameters and counteracting inflammatory processes [5,6]. However, their use in PCOS management needs deeper assessment regarding the effectiveness and the limits of their application. The authors, indeed, leave practical considerations out of their discussion, including the elevated costs of supplementation with ω3-PUFAs, their higher calorific value and the age of PCOS patients.

PCOS commonly involves insulin resistance and obesity, predisposing patients to cardiometabolic-related alterations such as dyslipidaemia, diabetes and hypertension [7,8]. Based on the evidence that PCOS management aims to alleviate symptoms depending on patient’s age and needs, and further considering that cardiovascular problems usually occur after 40 years of age in PCOS women [7], the administration of ω3-PUFAs is not generalizable to all types of PCOS patients. Even though previous studies reported promising results by using ω3-PUFA-enriched diets in PCOS patients on some cardiometabolic risk factors [9], they could not be reproduced systematically [10], and long-term analyses have not been conducted yet [11]. Therefore, according to the available evidence, ω3-PUFAs supplementation should be limited to women over 40 years of age that have already exhibited inflammatory and cardiovascular symptoms related to PCOS. Several lines of evidence further revealed that supplementation with ω3-PUFAs in PCOS patients failed to improve other symptoms related to the disorder, such as body weight and hip circumference, number of ovarian follicles, size of ovary, menstrual bleeding and hirsutism score [12].

On the contrary, based on the available information, supplementation with ω3-PUFAs in younger patients seems unnecessary [13].

Furthermore, EPA:DHA ratio is a crucial aspect of ω3-PUFA administration. Indeed, the use of different ratios correlates with different therapeutic benefits. In particular, an EPA:DHA ratio of 2:1 generally appears to be the most effective against inflammatory processes and cardiometabolic alterations [14]. According to studies in adults, the recommended minimum dose for combined EPA:DHA administration is 500 mg/day, reaching 2000–4000 mg/day in patients with a recent myocardial infarction or exhibiting altered triglycerides blood concentrations [15]. Based on this evidence, supplementation with ω3-PUFAs in PCOS should be carefully evaluated, considering the difficulties in finding adequate sources of ω3-PUFAs and the high costs of a ω3-PUFA-enriched diet [16]. 

Furthermore, the use of ω3-PUFAs is not devoid of adverse effects that need to be taken into account, including gastrointestinal symptoms such as heartburn and nausea. Notably, guidelines recommend avoiding EPA administration in pregnant women, while the intake of DHA and arachidonic acid (AA) is encouraged during pregnancy [17]. Indeed, EPA can compete with AA for the enzymes responsible for the formation of eicosanoids (cyclooxygenase, lipoxygenase) [18], which are crucial for neural foetal development [19]. Moreover, being fatty acids, ω3-PUFAs exhibit a higher calorific value compared to other dietary supplements [20]. Accordingly, supplementation with ω3-PUFAs in PCOS women, especially in those with obesity, should be mostly avoided [21]. 

In conclusion, even though ω3-PUFAs show several beneficial effects on human health, their administration in PCOS should be carefully assessed. Positive effects of supplementation with ω3-PUFAs are limited to a small portion of PCOS patients; in particular, those over 40 years of age and with cardiometabolic problems. Furthermore, daily administration of EPA and DHA is costly, and it deserves proper monitoring due to the possible adverse effects.

## Data Availability

Not applicable.
